# Degradation of MYC by the mutant p53 reactivator drug, COTI-2 in breast cancer cells

**DOI:** 10.1007/s10637-023-01368-1

**Published:** 2023-05-26

**Authors:** Minhong Tang, John Crown, Michael J Duffy

**Affiliations:** 1 UCD School of Medicine, Conway Institute of Biomolecular and Biomedical Research, University College Dublin, Dublin, Ireland; 2 Department of Medical Oncology, St Vincent’s University Hospital, Dublin, Ireland; 3 Clinical Research Centre, St Vincent’s University Hospital, Elm Park, Dublin, D04 T6F4 Ireland

**Keywords:** MYC, p53, COTI-2, Proteasome, Degradation, Breast cancer

## Abstract

TP53 (p53) and MYC are amongst the most frequently altered genes in cancer. Both are thus attractive targets for new anticancer therapies. Historically, however, both genes have proved challenging to target and currently there is no approved therapy against either. The aim of this study was to investigate the effect of the mutant p53 reactivating drug, COTI-2 on MYC. Total MYC, pSer62 MYC and pThr58 MYC were detected using Western blotting. Proteasome-mediated degradation was determined using the proteasome, inhibitor MG-132, while MYC half-life was measured using pulse chase experiments in the presence of cycloheximide. Cell proliferation was assessed using the 3-(4,5-dimethylthiazol-2-yl)-2,5-diphenyltetrazolium bromide (MTT) method. Treatment of 5 mutant p53 breast cancer cell lines with COTI-2 resulted in dose-dependent MYC degradation. Addition of the proteasome inhibitor, MG132, rescued the degradation, suggesting that this proteolytic system was at least partly responsible for the inactivation of MYC. Using cycloheximide in pulse chase experiments, COTI-2 was found to reduce the half-life of MYC in 2 different mutant p53 breast cancer cell lines, i.e., from 34.8 to 18.6 min in MDA-MB-232 cells and from 29.6 to 20.3 min in MDA-MB-468 cells. Co-treatment with COTI-2 and the MYC inhibitor, MYCi975 resulted in synergistic growth inhibition in all 4 mutant p53 cell lines investigated. The dual ability of COTI-2 to reactivate mutant p53 and degrade MYC should enable this compound to have broad application as an anticancer drug.

**Supplementary Information:**

The online version contains supplementary material available at 10.1007/s10637-023-01368-1.

## Introduction

*TP53 (p53)* and c-*MYC* (henceforth referred to as *MYC*) are amongst the most frequently altered driver genes in cancer [1–5]. Thus, *TP53* is the most frequently mutated gene in both primary and metastatic cancers [1, 2], while *MYC* is believed to be deregulated/overexpressed in up to 70% of all cancers [3–5]. Overall, MYC may be the most frequently dysfunctional driver gene in cancer [3–5]. Dysfunction promotes cancer formation and progression using 2 main mechanisms, i.e., *via* a cell intrinsic process in which MYC promotes growth of malignant cells and an extrinsic process in which the oncoprotein promotes evasion of host cell immunity [3–5].

Recent data suggests that MYC and p53 interact in promoting the growth of several different cancer types [6–12]. In particular, several studies have shown that *TP53* dysfunction (mutation or loss) enhanced the activation and stability of MYC [9–12]. One of the mechanisms by which mutant p53 enhances MYC stability appears to be *via* inhibition of the proteasome system which controls the cellular degradation of MYC [11, 12].

Since p53 and MYC are frequently altered in cancer, they are both highly attractive targets for new treatments against the disease. However, both mutant p53 and MYC have historically been challenging to successfully target, as both proteins lack a suitable hydrophobic crevice for high-affinity binding of potential low molecular weight drugs [13]. Furthermore, both proteins are predominantly present in the cell nucleus and therefore cannot be readily accessed by large molecular therapeutics such as conventional monoclonal antibodies [13].

Despite these difficulties, several promising compounds targeting mutant p53 or MYC have recently been described. These include the mutant p53 correcting compounds such as eprenetapopt (APR-246), COTI-2, PC14586 and arsenic trioxide which convert mutant p53 back to a protein with multiple wild-type properties [14, 15] and OmoMYC which prevents MYC from attaching to its cognate DNA binding sites [16, 17]. Indeed, all these compounds have recently progressed to clinical trials [14–17].

One of the compounds shown to reactivate mutant p53 into a protein with wild-type properties is the thiosemicarbazone, dubbed COTI-2 [N-(6,7-dihydro-5 H-quinolin-8-ylideneamino)-4-pyridin-2-ylpiperazine-1-carbothioamide] [18, 19]. Consistent with its ability to recover the wild-type configuration of mutant p53, treatment with COTI-2 has been shown to result in wild-type p53 DNA binding and normalized wild-type p53 target gene expression [19]. Furthermore, COTI-2 has been shown to reduce cancer cell proliferation or induce cancer cell apoptosis in diverse preclinical models [18–21].

Since mutant p53 stabilizes MYC [11, 12], reactivation of the mutant protein to its wild-type configuration with drugs such as COTI-2 might be expected to result in MYC degradation. The aim of this study was thus to investigate if treatment of breast cancer cell lines containing mutant *TP53* with COTI-2 leads to MYC degradation. As triple-negative breast cancer (TNBC) is the subtype of breast cancer with the worst outcome and the subtype most urgently in need of new treatments [22], our primary focus was on cell lines derived from this form of the disease.

## Materials and methods

### Cell lines and reagents

The origin and maintenance of the breast cancer cell lines used was as previously described [23, 24]. COTI-2 was purchased from Stratech Scientific Ltd, UK, MYCi 975 (HY-12,960) from MedChemTronica (HY-129,601), Bergkällavägen 37 C, 192 79 Sollentuna, Sweden and both MG-132 and 3-(4,5-dimethylthiazol-2-yl)-2,5-diphenyltetrazolium bromide (MTT) form Sigma-Aldrich, Ireland.

### Western blot analysis

Protein was extracted from COTI-2 treated cells as described previously [24]. Extracted proteins (50 µg) were then separated on a 10% handmade SDS-PAGE or precast Bolt Bis-Tris gels (Invitrogen) and transferred onto a nitrocellulose (NC) (Cytiva) or PVDF membrane (Cytiva) [25]. Membranes were then blocked in 5% bovine serum albumin (BSA) or milk TBST for one hour at room temperature, followed by staining with one of the following primary antibodies, anti-c-MYC (Abcam, ab32072), anti-c-MYC (phosphoS62) (Abcam, ab185656) or anti-c-MYC (phosphoT58) (Cell Signalling Technology, #46650S). GAPDH (PROTEINTECH EUR, 6000-4-Ig) was used as loading control. After triple washing (10 min each) in TBST, membranes were immersed in 5% BSA or milk TBST containing HRP-conjugated secondary antibody (Santa Cruz) for 1.5 h at room temperature, followed by another 3 washes. Targeted proteins were probed by Super Signal chemiluminescence (ECL) (Thermo Fisher Scientific) and visualized using the Odyssey Imaging System (LI-COR Biosciences). Band intensities were quantified by densitometry using Image J software.

### Measurement of proteasome inhibition and determination of MYC half-life

To detect proteasome inhibition, breast cancer cell lines were treated with 20 µM MG-132 for 3 h, followed by another 3 h incubation with 2 µM COTI-2 or DMSO. The relative MYC protein levels were analyzed by Western blotting. For measuring MYC protein half-life, breast cancer cell lines were incubated with 2 µM COTI-2 or DMSO for 3.5 h and then treated with cycloheximide (30 µg/ml). Cells were then harvested followed, by Western blotting and staining for MYC.

### RNA extraction and real time PCR

RNA extraction and cDNA preparation were as previously described [24]. MYC primers were purchased from Sino Biological Inc. GAPDH (Sigma-Aldrich) was used as a housekeeping-gene control. The amplification process was carried out as recommended by Sino Biologicals Inc for the Roche Light Cycler 480. Changes in gene expression were analyzed using the Delta-Delta Ct (ddCt) method.

### Cell proliferation assays

Cell growth was assessed using 3-(4,5-dimethylthiazol-2-yl)-2,5-diphenyltetrazolium bromide (MTT) as previously described [23, 24].

### Statistical analysis

Microsoft Excel 2016 was used for the initial analysis of all the raw data. Graph Pad Prism 5 was used to graph the calculated data points and calculate statistical values. The significance of data was evaluated using the Student’s unpaired, two-tailed t-test. Combination indices (CI values) for co-treatment of cells with COTI-2 and MYCi975 were calculated using CalcuSyn software (Biosoft) [29]. CI values < 1 at 50% inhibition was used to indicate synergism [26].

## Results

### Effect of COTI-2 on MYC degradation in breast cancer cell lines

**Fig. 1:**
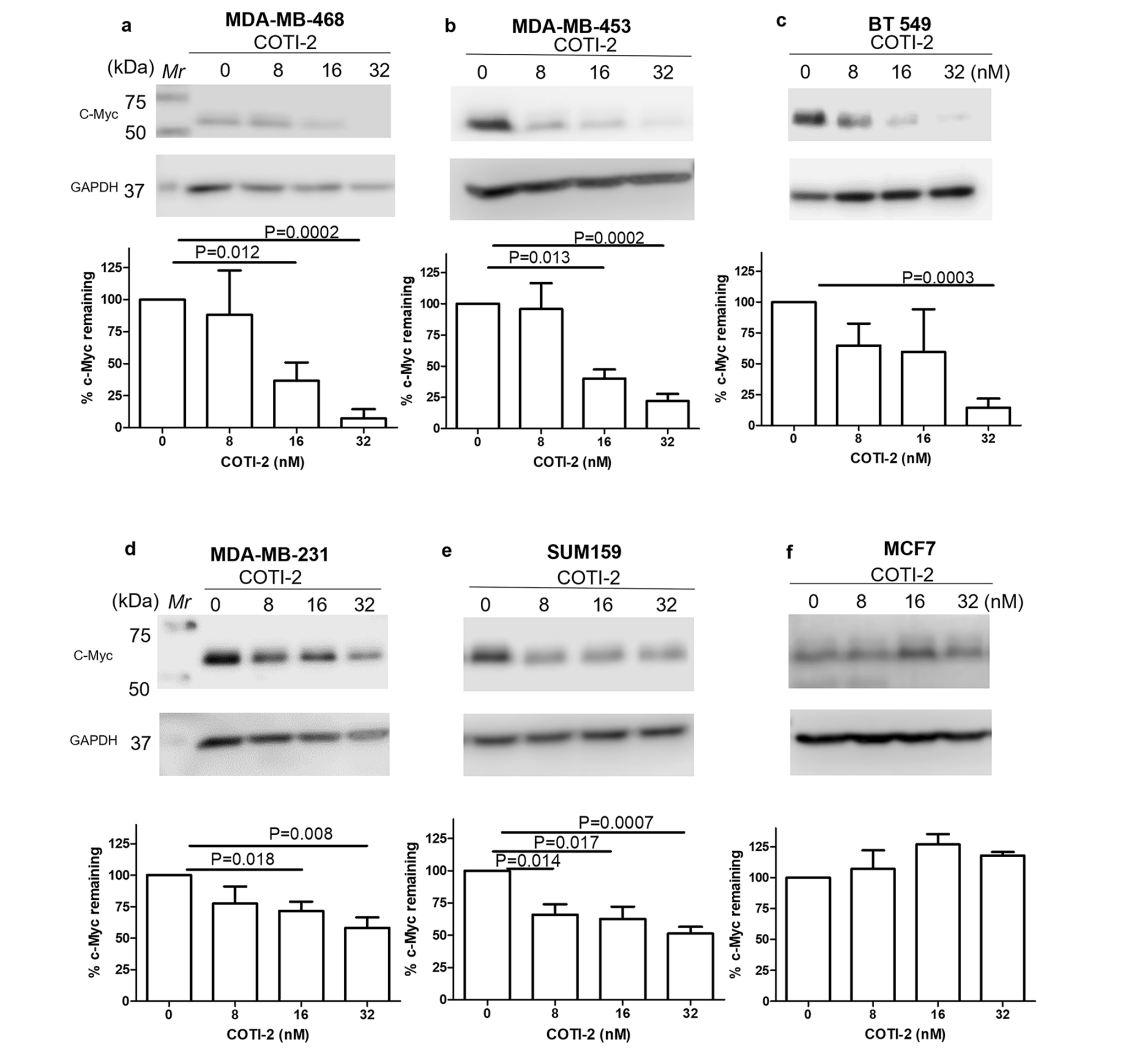
Effect of COTI-2 on degradation of MYC. (**a**) MDA-MB-468, (**b**) MDA-MB-453, (**c**) BT549, (**d**) MDA-MB-231, (**e**) SUM159 and (**f**) MCF7. Cells were incubated with different concentrations of COTI-2 for 48 h. Cell lysates were assessed by Western blotting. GAPDH was probed as loading control. Expression fold changes and % of MYC remaining were then calculated and graphed using GraphPad Prism 5. Data plotted are means ± S.E.M (n = 3) and evaluated using the Student’s unpaired, two-tailed t-test

**Fig. 2:**
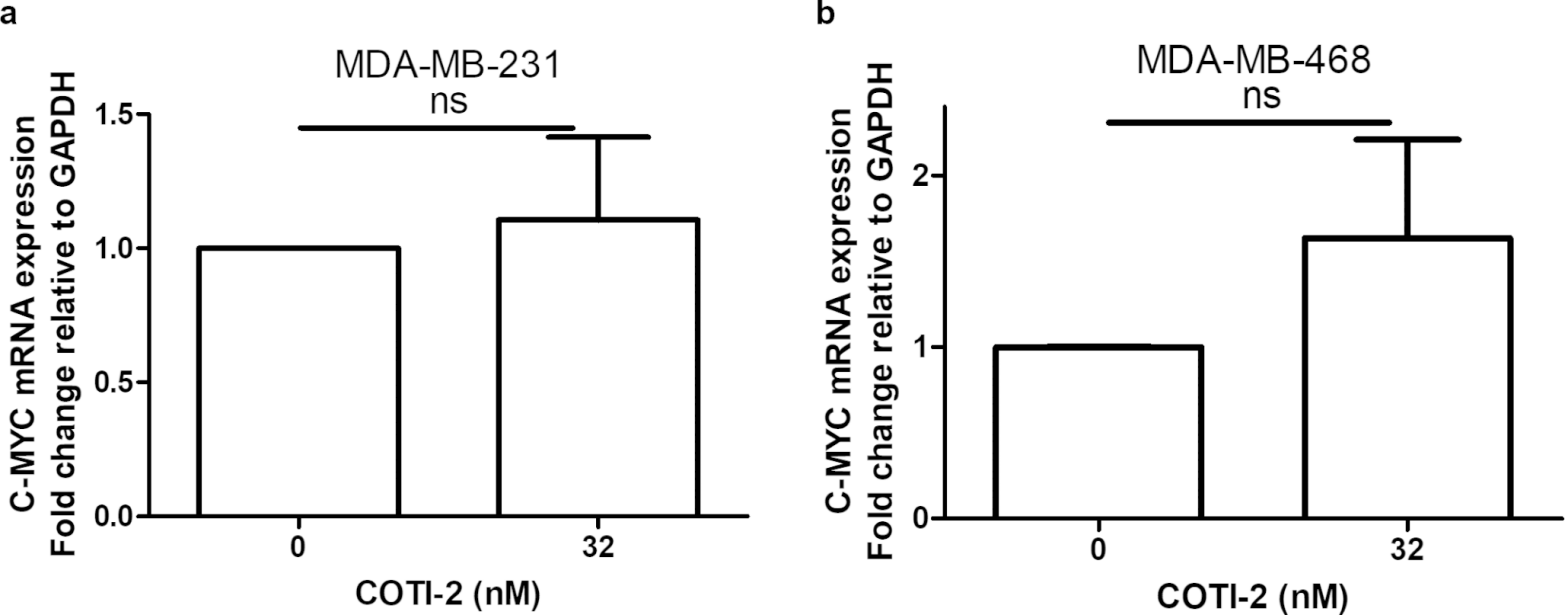
Effect of COTI-2 on expression of MYC mRNA. (**a**) MDA-MB-231 and (**b**) MDA-MB-468 cells were incubated with DMSO or 32 nM of COTI-2 for 48 h. mRNA expression level were determined by RT-PCR. Data were calculated and graphed using GraphPad Prism 5. Data plotted are means ± S.E.M (n = 3) and evaluated using the Student’s unpaired, two-tailed t-test

As previous studies showed that mutant p53 enhanced the stability of MYC [11, 12], reactivation of the mutant protein to a form with wild-type functionality might be expected to lead to MYC destabilization. To test this possibility, we investigated the effects of the mutant p53 reactivator compound, COTI-2 on MYC degradation. Consistent with its ability to reactivate mutant p53 to a wild-type-like form, treatment with COTI-2 resulted in MYC degradation in all 5 of the investigated cell lines containing mutant p53 (Fig. 1a-e). In contrast to our findings with the mutant p53-containing cell lines, COTI-2 had no effect on the degradation of MYC in the p53 wild-type cell line, MCF-7 (Fig. 1f). Theoretically, the decreased levels of MYC protein observed might also have been due to enhanced MYC mRNA degradation or other mechanisms at the transcription levels. However, by measuring mRNA using RT-PCR, we found no significant effect of COTI-2 on this molecular species (Fig. 2).

### Attempt to identify mechanisms by which COTI-2 mediated degradation of MYC

**Fig. 3:**
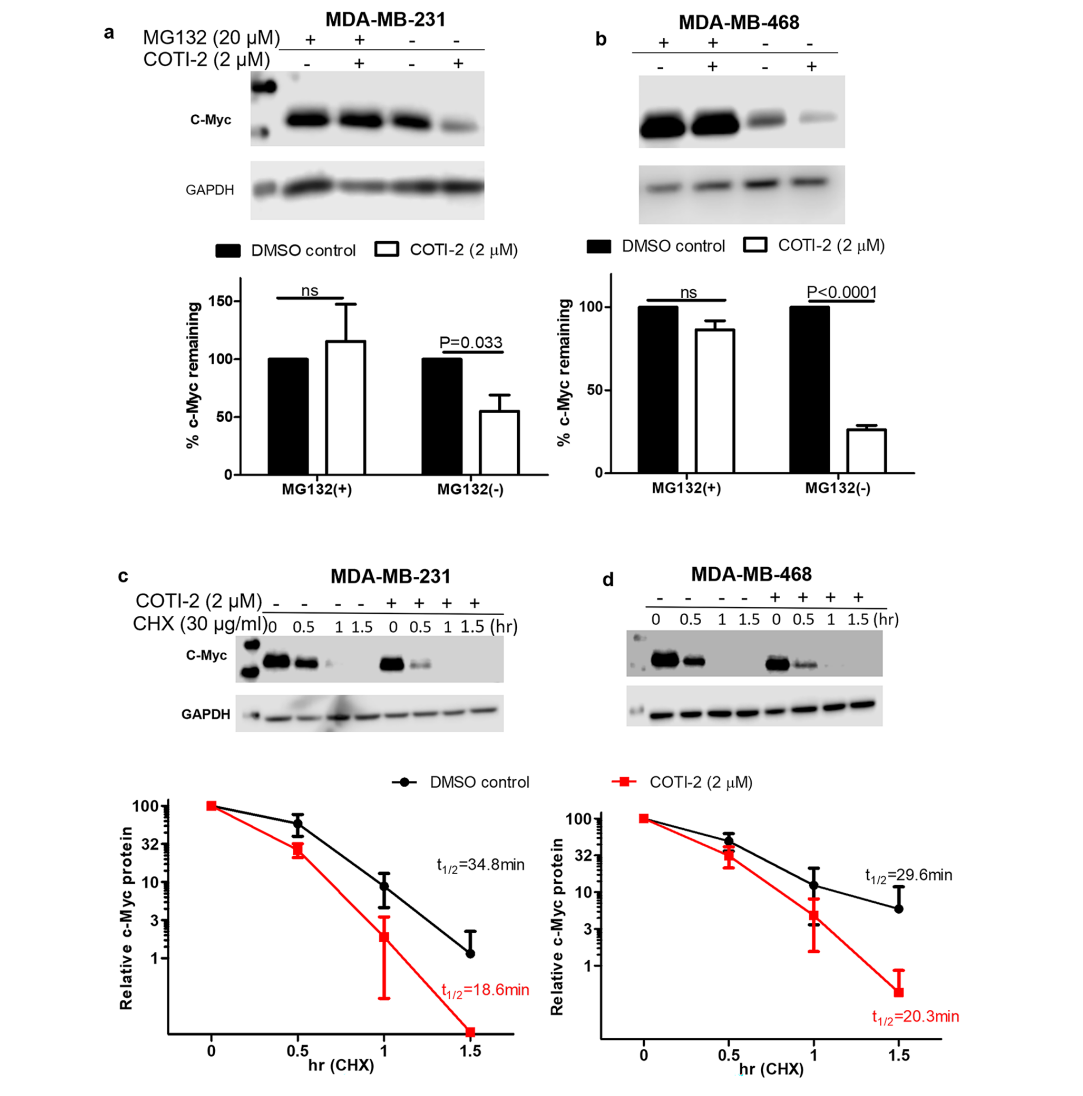
Effect of COTI-2 on the proteasomal degradation of MYC. (**a**) MDA-MB-231 and (**b**) MDA-MB-468 cells were incubated with 20 µM of MG-132 for 3 h, followed by 3 h incubation with DMSO or 2 µM of COTI-2, before harvesting. (**c**) MDA-MB-231 and (**d**) MDA-MB-468 were treated with DMSO or 2 µM of COTI-2 for 3.5 h, followed by incubation with CHX (30 µg/ml). Treated cells were then harvested at the indicated time points. Degradation of MYC was visualized by Western blotting. Data were calculated and graphed using GraphPad Prism 5. Data plotted are means ± S.E.M (n = 3) and evaluated using the Student’s unpaired, two-tailed t-test

As the proteasome network is the best-known intracellular mechanism for the breakdown of intracellular proteins including MYC [27], we initially investigated if this system was involved in MYC degradation. To do this, we incubated cells with the proteasome inhibitor, MG-132. As shown in Fig. 3a and b, incubation with MG-132 decreased the COTI-2-induced reduction of MYC in the 2 cell lines tested, suggesting that the proteasome system is at least partially contributing to the reduced MYC protein levels.

To determine the effect of COTI-2 on the cellular half-life of MYC, we performed pulse chase experiments using the protein synthesis inhibitor, cycloheximide (Fig. 3c and d). As can be seen, COTI-2 decreased the MYC protein half-life from 34.8 to 18.6 min in MDA-MB-231 cells and from 29.6 to 20.3 min in MDA-MB-468 cells.

### Effect of COTI-2 on phosphorylation of MYC

**Fig. 4:**
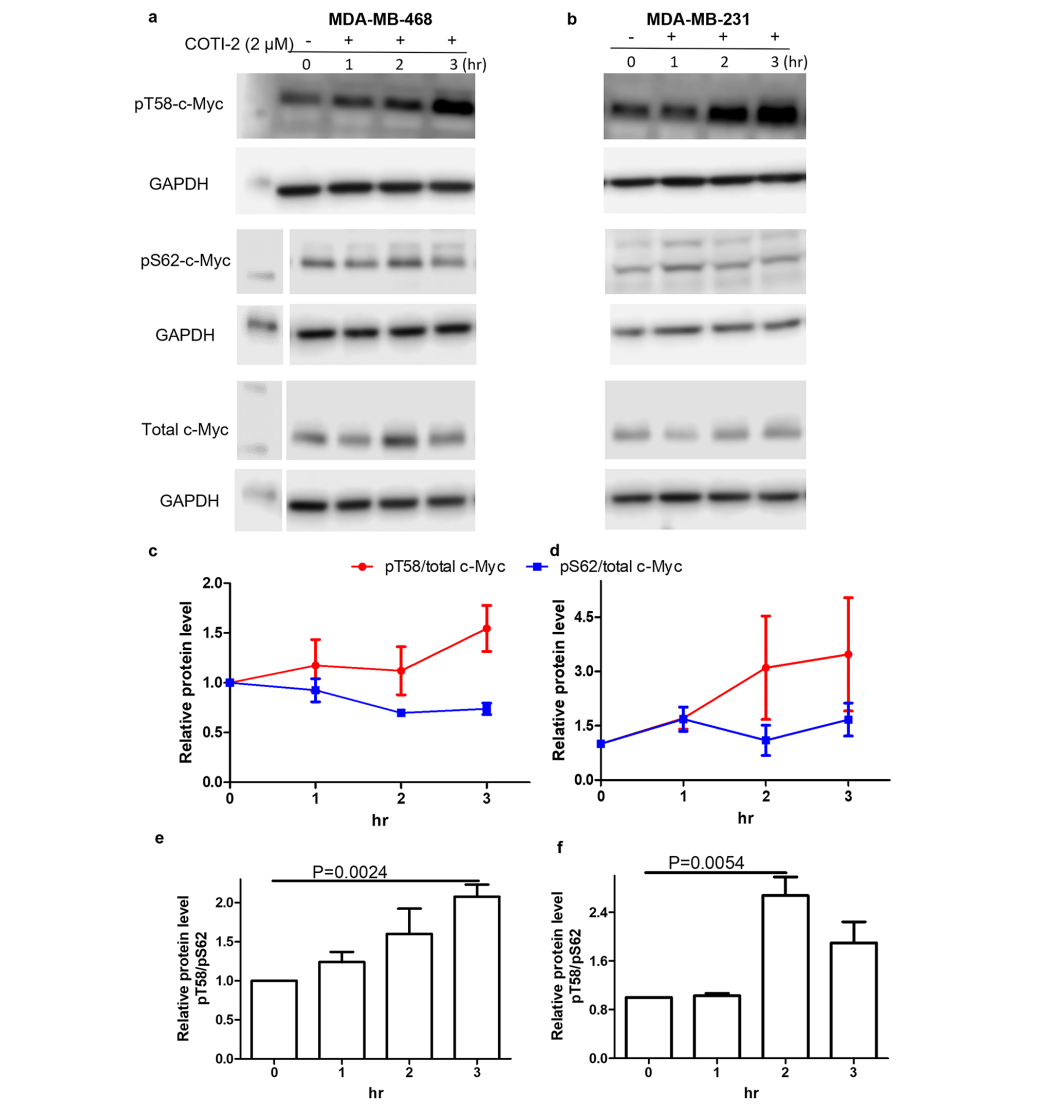
Effect of COTI-2 on phosphorylation of MYC. (**a**) MDA-MB-468 and (**b**) MDA-MB-231 cells were incubated with 2 µM of COTI-2. Cells were harvested at the indicated time points. Cell lysates were determined by Western blotting using antibodies against pT58 MYC, pS62 MYC, and total MYC. GAPDH was probed as loading control. **c**,**d**,**e** **f**) Effect of time of incubation with COTI-2 on relative levels of pT58 versus pS62 in MDA-MB-468 (**c**,**e**) MDA-MB-468 cells (**d**,**f**). Data was calculated and graphed using GraphPad Prism 5. Data plotted are means ± S.E.M (n = 3)

One of the best-established mechanisms leading to the proteasome-mediating degradation of the MYC protein involves sequential phosphorylation, initially at serine 62 (pS62) catalyzed by kinases, such as ERK and/or CDK2. This in turn is followed by phosphorylation on threonine 58 (pT58) mediated by GSK3β [28, 29]. The pT58 form then undergoes ubiquitination in the presence of E3 ubiquitin ligases such as FBXW7 which ultimately results in degradation by the proteasome system. To establish if MYC pT58 or pS62 levels were altered by treatment with COTI-2, we performed Western blotting with specific antibodies against these different phosphorylated forms of MYC. Consistent with the above-mentioned mechanism of MYC degradation, COTI-2 was found to increase the pT58 phosphorylated form of MYC but had little effect on the pS62 phosphorylation form or total MYC (Fig. 4a and b). Furthermore, COTI-2 significantly increased the relative levels of the pT58 form versus the pS62 form over time, i.e., over 3 h of treatment in the MDA-MB-468 cell line and over 2 h in MD-MBA-231 cells. In contrast to our findings with the pT58 form of MYC, COTI-2 had no effect on total MYC levels following 3 h of incubation (Fig. 4c-f). Although there was no degradation of total MYC within the first 3 h of COTI-2 treatment, degradation started to occur after 4 h (Fig. 1 Suppl).

### Effects of combined treatment with COTI-2 and the MYC inhibitor, MYCi975 on cell proliferation

**Fig. 5:**
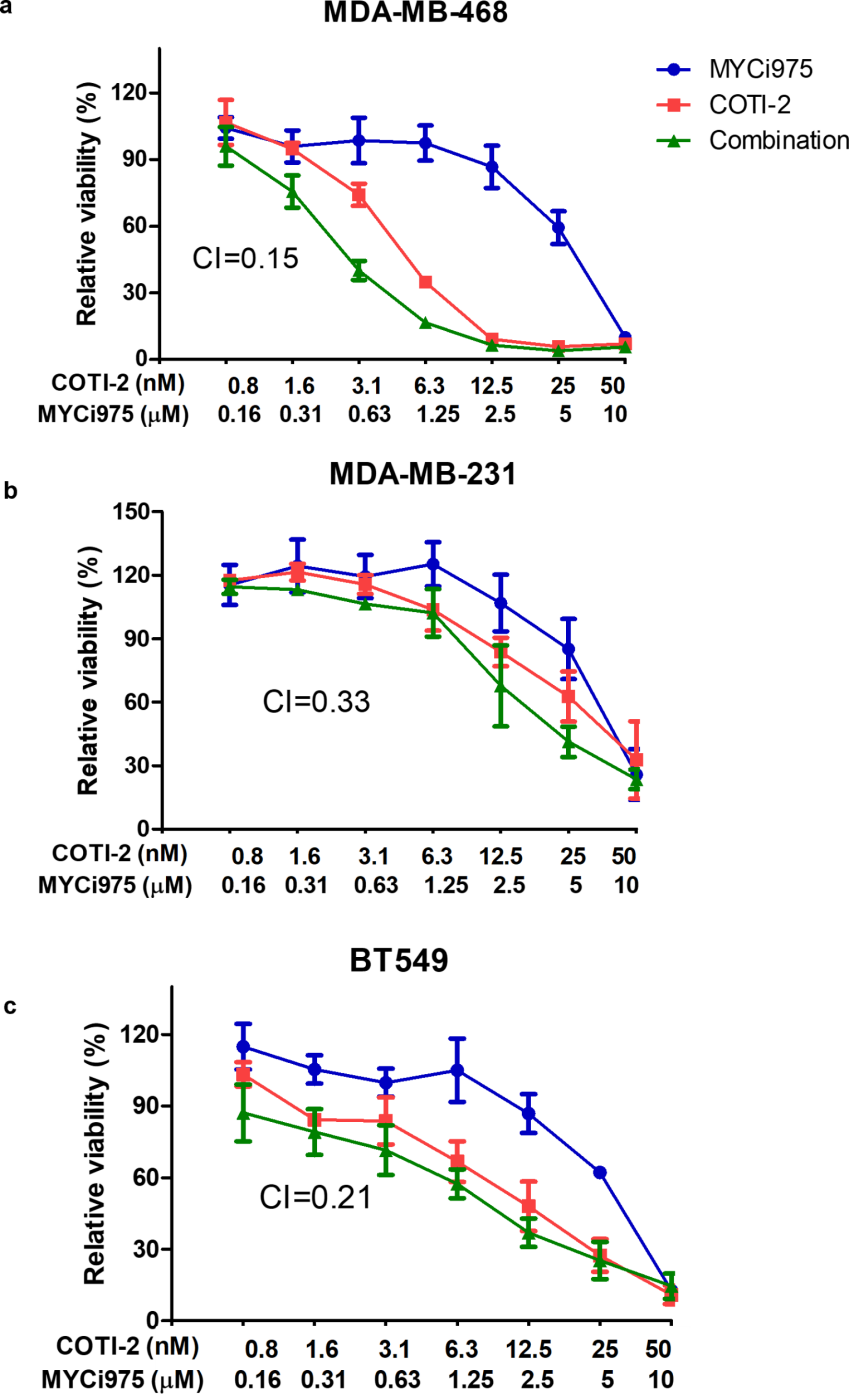
Effects of combined treatment with COTI-2 and MYCi975 on cell proliferation (**a**) MDA-MB-468, (**b**) MDA-MB-231 and (**c**) BT 549 cells were incubated with various concentrations of COTI-2 and MYCi975 for five days. MTT assays were then carried to detect cell proliferation. Combination index (CI) values were calculated using Compusyn software. CI values < 1 indicate drug-synergy. Data were calculated and graphed using GraphPad Prism 5. Data plotted are means ± S.E.M (n = 3)

Combined treatment with multiple drugs is increasing being used for patients with cancer. To increase the anti-proliferative impact of COTI-2, we combined it with the MYC inhibitor, MYCi975 [24, 30]. When 2 different drugs are combined, the end results may be antagonism, no additional effect, an additive effect or synergism. To differentiate between these 4 outcomes, we used the Chou-Talalay method to calculate the combination indices (CI) values for the combined treatment [26]. As the CI values for the combined treatments were < 1 in all the 3 cell lines investigated (Fig. 5), we conclude that co-treatment with the mutant p53 reactivator drug, COTI-2 and the MYC inhibitor, MYCi975 result in synergistic growth inhibition in all 3 cell lines investigated.

## Discussion

As mentioned in the Introduction above, MYC remains one the most attractive oncoprotein remaining to be exploited for therapeutic value in cancer patients [31, 32]. Indeed, several anti-MYC inhibitors are currently undergoing preclinical studies [31, 32] and at least one, i.e., Omomyc, has been investigated in an early clinical trial [33]. Interestingly, similar with some of the MYC inhibitors currently under study such as Omomyc [34], MYCi975 and MYCMI-6 [24, 30], we show that COTI-2 can also degrades MYC. However, in contrast to the classical MYC inhibitors which degraded MYC protein at µM concentrations, COTI-2 caused its effects at low nM concentrations (< 30 nM). Assuming COTI-2 could eventually be used to treat patients, this ability to degrade MYC at low concentration might be expected to have benefits with respect to reduced drug-induced toxicity.

Based on our finding with the proteasome inhibitor, MG-132, COTI-2 appeared to mediate MYC degradation, at least in part, by activating the proteasome system. This finding is consistent with previous studies which suggested that mutant p53 prevented MYC degradation by inhibition of the proteasome system [11, 12]. Indeed, we found that COTI-2 enhanced levels of the pT58 phosphorylated form of MYC but had little effect on phosphorylation at the pS62 site. Incidentally, the MYC inhibitor, MYCi371 (an anlog of MYCi975) has also been reported to increase levels of the pT58 form of MYC [30]. Similar to our results with COTI-2, it also failed to alter levels of the pS62 form [30]. It is of interest that 2 other compounds reported to reactivate mutant p53, were also found to degrade MYC, i.e., APR-246 [35, 36] and arsenic trioxide [37].

Degradation of cancer driver oncoproteins is emerging as a promising new approach to target cancer driver proteins that historically have proved difficult to inhibit. Amongst the strategies undergoing extensive investigation for this purpose are PROTACS and molecular glues [38]. Indeed, recently, a novel molecular glue dubbed WBC100 was also found to degrade MYC by activating the proteasome system [39]. Degradation of MYC led to tumor regression in multiple xenograft mouse models.

Theoretically, drugs capable of simultaneously transforming mutant p53 to a form with wild-type properties and degrading MYC, such as COTI-2, might be expected to have a broad application in cancer treatment. In previous studies we showed that treatment with either the mutant p53 reactivating drugs, COTI-2 [18] or the MYC inhibitor, MYCi975 [24] resulted in decreased tumor cell proliferation and induction of apoptosis in mutant p53 breast cancer cell lines. Of potential clinical significance was our observation that both compounds were significantly more potent in tripe-negative (TN) than in non-TN cell lines [18, 24]. Here, we show that the combination of these 2 compounds led to synergistic growth inhibition in the 3 mutant p53 TN breast cancer cell lines investigated. COTI-2 may thus be a potential new therapy for patients with TN breast cancer, the subtype of breast cancer in which new therapies are most urgently required. Yet, a further incentive for investigating mutant COTI-2 as a potential therapy for TN breast cancer is that p53 is mutated in approximately 80% [14, 15] and MYC is amplified in up to 60% of patients with this molecular form of breast cancer [40, 41].

A limitation of our work is that it was mostly confined to cancer cell lines belonging to a molecular subtype of breast cancer, i.e., TNBC. Our finding may thus not be relevant for other molecular subtypes of breast cancer or indeed for other cancer types. Clearly, our results require confirmation using in vivo model systems. If confirmed in such models, without major toxicity, it would provide additional preclinical evidence for further investigating COTI-2 in clinical trials. Although multiple preclinical studies have shown that COTI-2 has anticancer activity [18–21], there is still no published evidence that the drug possesses clinical activity. A phase I clinical trial to assess the safety and tolerability of COTI-2 in patients with advanced or recurrent malignancies, however, has been carried out (ClinicalTrials.gov Identifier: NCT0243362). Early interim data from the trial suggested no major toxicity (https://www.globenewswire.com/news-release/2019/05/08/1819580/0/en/Cotinga-Pharmaceuticals-Releases-Early-Interim-Data-of-Phase-1b-2a-Combination-Trial-of-COTI-2-in-Solid-Tumors.html.

Before concluding this manuscript, we should mention that COTI-2 was reported to have additional activities that were apparently independent of mutant p53 reactivation [19]. These activities included induction of DNA damage, promotion of replication stress, activation of AMPK and inhibition of the mTOR pathway. While theoretically, some of these actions could lead to MYC degradation, based on current knowledge, it is a difficult to identify a mechanism by which they could do so.

In conclusion, we show that the mutant p53 reactivator compound, COTI-2 degraded MYC in mutant p53 breast cancer cells. Thus, COTI-2 has activity against 2 of the most frequently altered cancer driver genes. If confirmed, this finding may have major implications for COTI-2 as an anti-cancer drug.

## Electronic supplementary material

Below is the link to the electronic supplementary material.

Supplementary Material 1

## Data Availability

All data generated or analysed during this study are included in this published article and its supplementary information files.
