# Supplementary figures and images for: Degradation of MYC by the mutant p53 reactivator drug, COTI-2 in breast cancer cells
Source: Invest New Drugs. 2023 May 26;41(4):541–50. doi: 10.1007/s10637-023-01368-1 (PMC10447602; doi:10.1007/s10637-023-01368-1)

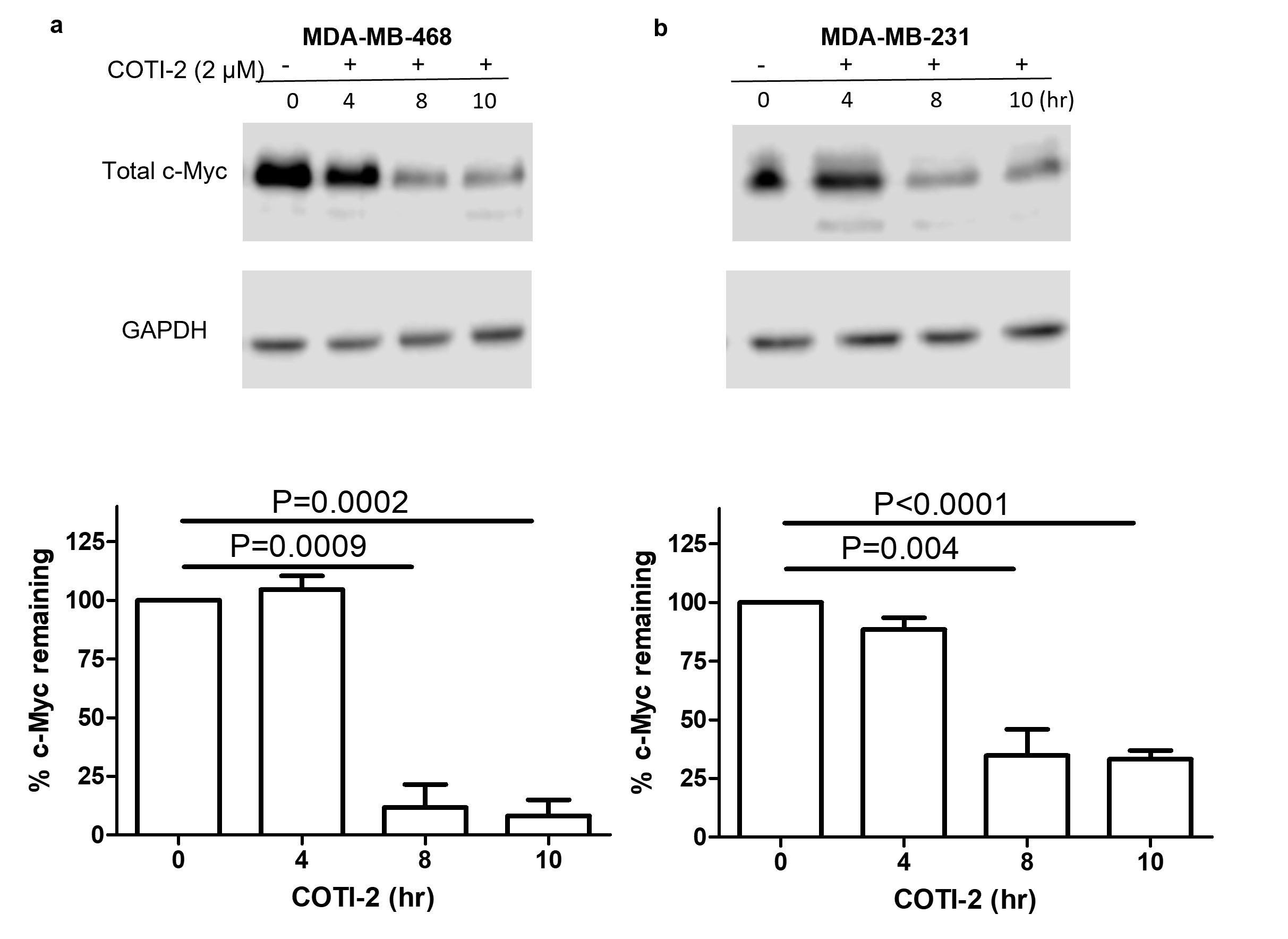

Supplement: Supplementary file 1 — Supplementary Material 1 [file 10637_2023_1368_MOESM1_ESM.png]
